# Design of a cross-sectional study on physical fitness and physical activity in children and adolescents after burn injury

**DOI:** 10.1186/1471-2431-12-195

**Published:** 2012-12-20

**Authors:** Laurien M Disseldorp, Leonora J Mouton, Tim Takken, Marco Van Brussel, Gerard IJM Beerthuizen, Lucas HV Van der Woude, Marianne K Nieuwenhuis

**Affiliations:** 1 Center for Human Movement Sciences, University of Groningen, University Medical Center Groningen, PO Box 196, 9700 AD, Groningen, The Netherlands; 2 Child Development and Exercise Center, Wilhelmina Children’s Hospital, University Medical Center Utrecht, Utrecht, The Netherlands; 3 Association of Dutch Burn Centers, Martini Hospital, Groningen, The Netherlands; 4 Center for Rehabilitation, University of Groningen, University Medical Center Groningen, Groningen, The Netherlands

**Keywords:** Burns, Outcome assessment, Child, Fatigue, Quality of Life

## Abstract

**Background:**

Burn injuries have a major impact on the patient’s physical and psychological functioning. The consequences can, especially in pediatric burns, persist long after the injury. A decrease in physical fitness seems logical as people survive burn injuries after an often extensive period of decreased activity and an increased demand of proteins leading to catabolism, especially of muscle mass. However, knowledge on the possibly affected levels of physical fitness in children and adolescents after burn injury is limited and pertains only to children with major burns. The current multidimensional study aims to determine the level of physical fitness, the level of physical activity, health-related quality of life and perceived fatigue in children after a burn injury. Furthermore, interrelations between those levels will be explored, as well as associations with burn characteristics.

**Methods/design:**

Children and adolescents in the age range of 6 up to and including 18 years are invited to participate in this cross-sectional descriptive study if they have been admitted to one of the three Dutch burn centers between 6 months and 5 years ago with a burn injury involving at least 10% of the total body surface area and/or were hospitalized ≥ 6 weeks. Physical fitness assessments will take place in a mobile exercise lab. Quantitative measures of cardiorespiratory endurance, muscular strength, body composition and flexibility will be obtained. Outcomes will be compared with Dutch reference values. Physical activity, health-related quality of life and fatigue will be assessed using accelerometry and age-specific questionnaires.

**Discussion:**

The findings of the current study will contribute to a better understanding of the long-term consequences of burn injury in children and adolescents after burns. The results can guide rehabilitation to facilitate a timely and optimal physical recovery.

**Trial registration:**

The study is registered in the National Academic Research and Collaborations Information System of the Netherlands (OND1348800).

## Background

Worldwide, millions of people suffer from burn-related disabilities and disfigurements. In the Netherlands, each year about 550–750 people are admitted to a dedicated burn center, of which approximately 40% is younger than 18 years [1]. The survival rate in patients with –even very extensive- burn injuries increased over the last decades. As a result, attention in international burn care and research has shifted from mortality towards the life after burns. Nowadays, dedicated burn centers provide multidisciplinary care and rehabilitation programs, generally starting at admission, in order to optimize patient’s outcomes. Nevertheless, a relevant part of the burn population still shows long-term declined physical and/or psychological functioning and/or suboptimal quality of life after burns [2-7]. Especially consequences of pediatric burns may persist from childhood through adolescence into adulthood. As in burn care, burn research nowadays intends to optimize the clinical process and patients’ outcomes on both short and long term post burn. Hence, our research group recently initiated multidimensional research on outcomes after burns comprising physical fitness, physical activity, health-related quality of life (HRQoL) and fatigue. The current study in children and adolescents is part of this research line.

Physical fitness as well as physical activity in children and adolescents are nowadays widely publicised topics due to the increasing awareness of their importance for health and well-being and, on the other hand, to prevent disability and morbidity throughout life. In children after a severe burn injury physical fitness is diminished (see Disseldorp, Nieuwenhuis, Van Baar, & Mouton, 2011 for review [8]). This decrease in physical fitness seems logical as people survive burn injuries after an often extensive period of physiological assault, decreased physical activity and an increased demand of proteins leading to catabolism, especially of muscle mass. However, the findings of diminished physical fitness are primarily based on children with severe burns involving >40% total body surface area [TBSA], representing only <5% of all pediatric burn patients [1,9]. Knowledge regarding physical fitness for the general population of children and adolescents after burns is lacking.

Physical activity, e.g. in the form of exercise programs, can significantly contribute to the restoration of physical fitness in children and adolescents after severe burn injury (among others [10-13]. Furthermore, physical activity is associated with improvements in health-related quality of life [14]. Despite its known positive effects, physical activity in this population might be diminished. This could be due to the loss of physical fitness, but other barriers may play a role as well, such as disfigurement and socioeconomic factors. However, physical activity levels in daily life after pediatric burns have been overlooked in burn research thus far.

Concerning health-related quality of life (HRQoL) research showed that long-term limitations in HRQoL are experienced by >50% of Dutch and Flemish children after burns [3]. The impact on HRQoL is not surprising, as both the burn accident and the persisting consequences of the burns can impact one’s appearance and functioning on several domains (e.g. the physical, psychological and social domain). Associations between HRQoL and both physical fitness and activity are plausible, though not yet examined in this population.

Another factor that impacts an individual’s daily functioning as well as activities and HRQoL, is fatigue [15]. Fatigue is often experienced in the long-term after burn injury, according to communication with the Dutch Association of Burn Survivors [personal communications]. Until now no studies on fatigue after pediatric burn injury, nor on its probable associations with physical fitness, activity or HRQoL after burns have been published.

**Figure 1:**
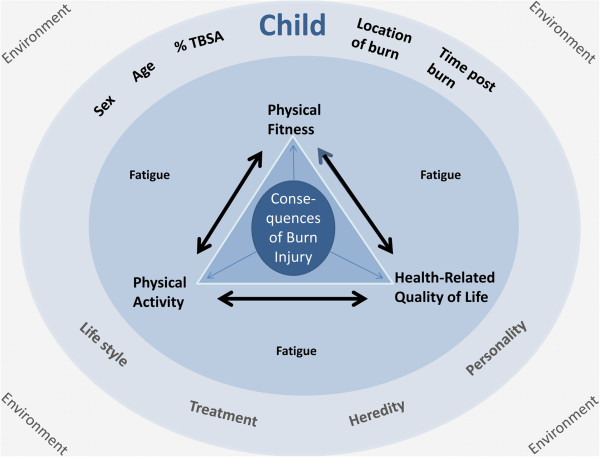
Conceptual model depicting the rationale of this multidimensional study.

The conceptual model that depicts the rationale of this study is shown in Figure 1. This model is partly based on the ideas of Bouchard et al. (1994), who emphasized the interrelations between physical activity, physical fitness and health (wellness) [16]. Also, concepts of the International Classification of Functioning, Disability and Health are taken into account [17]. Furthermore, both burn characteristics and individual characteristics are highlighted in the model, as we assume those to substantially influence the central aspects of this study, i.e. physical fitness, physical activity, HRQoL and fatigue.

The objective of the current paper is to describe the rationale and design of a cross-sectional study in children and adolescents after burn injury. The study aims to 1) determine the levels of physical fitness and physical activity in this group, also in comparison to reference values; 2) assess HRQoL and fatigue; and 3) explore what associations exist between fitness, activity, HRQoL and fatigue, taking individual (burn-related) characteristics into account. The results of the study will enlarge our understanding of the physical consequences of burn injury. Subsequently, this can lead to an adaptation of rehabilitation protocols to facilitate a timely and optimal recovery of future pediatric burn patients.

## Methods

### Design

In this cross-sectional descriptive study the levels of physical fitness and physical activity in children and adolescents after burn injury will be determined and compared with Dutch reference values. This design is chosen to gain insight in the physical consequences of burn injury in a group of subjects varying in extent of burn, time post burn and age, accomplished within a study period of one year.

### Setting

This study is a collaboration between the Association of Dutch Burn Centers (Epidemiology & Registration; Clinical Research), Wilhelmina Children’s Hospital (Child Development and Exercise Center) and the University Medical Center Groningen (Center for Human Movement Sciences, University of Groningen).

To keep the inconvenience of participation low and enlarge the feasibility of the study, assessments will take place on personal arrangement. The use of a mobile exercise lab enables us to meet the subjects and his/her parents at any date and time that is convenient for them, near the subject’s home (or other location of choice). The mobile exercise lab is a specially equipped truck in which equipment for cardiopulmonary exercise testing is installed, e.g. a cycle ergometer and a mobile gas analysis system, as well as other testing equipment. This truck has been in use for several years for (clinical) exercise testing [18].

### Subjects

Eligible for this study are all children and adolescents aged from 6 up to and including 18 years with healed burns that had been admitted to one of the Dutch burn centers with 10% TBSA or more involved in the burn and/or had a length of stay of more than 6 weeks, see Table 1. The source population will be restricted to children and adolescents for whom the time post burn is between 6 months and 5 years, and discharge and/or reconstructive surgeries are at least 2 months ago at the time of the assessment. Extensive (pre-existing) comorbidity or (mental) disabilities and insufficient Dutch language proficiency are criteria for exclusion, see Table 1. Informed consent must be provided by all parents (or legal representatives) as well as by subjects up from 12 years of age before enrolment in this study; only for subjects aged 18 parental informed consent is not requested. Contra-indications for exercise testing, based on the Exercise Preparticipation Screening questionnaire or from consultation with the burn physician, may lead to exclusion on the cardiopulmonary exercise test. The Medical Ethical Committee of the University Medical Center Groningen approved this study (NL40183.042.12).

**Table 1:** Criteria for eligibility and exclusion of subjects

**Eligibility**	**Exclusion**
Admitted to Dutch burn center	Time post burn < 0.5 year or > 5 years
Burns ≥ 10% TBSA; or hospitalization ≥ 6 weeks	(Pre-existing) comorbidity or (mental) disabilities
Aged 6 up to and including 18 years	Insufficient Dutch language proficiency
	No signed informed consent
	

### Sample size

A precise sample size calculation is not possible as no data are available on a similar population, as this study is the first to assess physical fitness, physical activity, HRQoL and fatigue in such a population. Nevertheless, we made an estimation of the expected sample size. At the first inclusion date, August 1^st^, 2012, 56 children met the inclusion criteria as identified in the Dutch Burn Repository [1]. At the second inclusion date in the spring of 2013, it is estimated that 10 more children will be eligible. However, it is probable that not all eligible children can be included and, secondly, not all included children will be reached and/or will agree to participate. We expect to be able to assess about 30 subjects.

### Procedure

**Figure 2:**
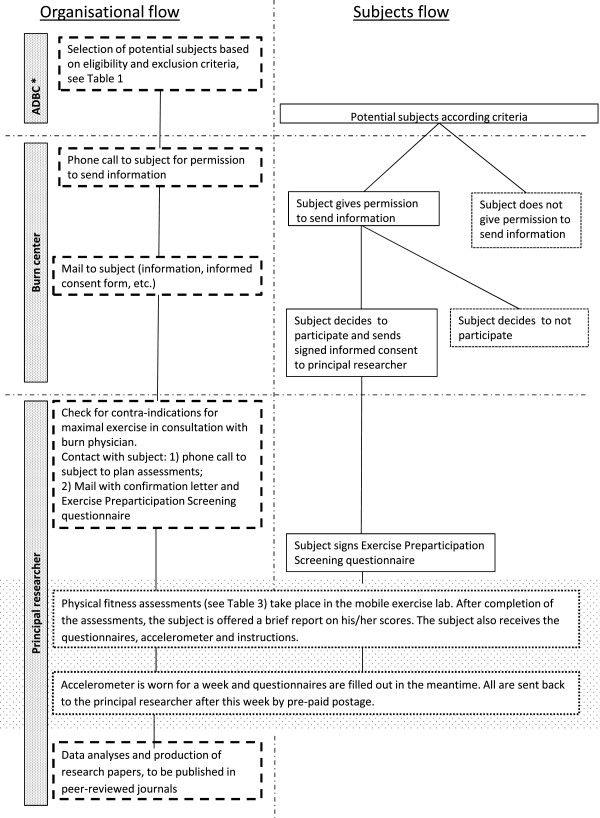
Chart of both organisational and subject flow in this study.

Inclusion and study procedures are depicted in Figure 2. Demographic and burn (treatment) characteristics, e.g. age, sex, extent of burn, location of burn, time post burn, presence of inhalation injury and the number of surgeries, will be documented for each subject. Further, subjects will be assessed once on physical fitness, as described below and shown in Table 2 and Figure 2. Estimated duration of the physical fitness assessment session is 1.5 h, including introduction, explanations and social talk. For the assessments of physical activity, HRQoL and fatigue the accelerometer and questionnaires, including instructions, will be handed out on the day of physical fitness assessment and taken home by the subjects, see Table 2 and Figure 2. The subjects are requested to wear the accelerometer for seven consecutive days, preferably starting the day after the fitness assessments, and return the questionnaires and accelerometer by mail (pre-paid postage) after this week.

**Table 2:** Overview of involvement in assessments and questionnaires given per subject age groups

**Age subject**		**Mobile exercise lab**	**Home based**			
		**Physical fitness**	**Physical activity**		**HRQoL**	**Fatigue**
		**Fitness assessments**	**Accelerometry**	**Activity Quest.**	**BOQ**	**PedsQL-MFS**
**6-7 years**	**Child**	√	√			CR 5-7
	**Parents**			PR 6-11	PR 5-18	PR 5-7
**8-11 years**	**Child**	√	√			CR 8-12
	**Parents**			PR 6-11	PR 5-18	PR 8-12
**12 years**	**Child**	√	√	CR 12-18	CR 11-18	CR 8-12
	**Parents**					PR 8-12
**13-18 years**	**Child**	√	√	CR 12-18	CR 11-18	CR 13-18
	**Parents**					PR 13-18

*HRQoL*, health-related quality of life; *BOQ*: Burns Outcomes Questionnaire; *PR*, parent report; *CR*, child report; *PedsQL-MFS*, PedsQL Multidimensional Fatigue Scale.

**Table 3:** Overview of study outcomes with corresponding instruments and units of measurement

**Construct**	**Component**	**Variable** (Instrument)
		*Parameter [unit]*
**Physical fitness**	**Cardiorespiratory endurance**	**Aerobic exercise capacity** (Cycle ergometer + gas analyzer)
		*VO2*_*peak*_*[ml·min*^*-1*^*]*
		*VO2*_*peak*_*relative for weight [ml·kg*^*-1*^*·min*^*-1*^*]*
		*%HR01 [%]*
		*%HR02 [%]*
		*WR*_*peak*_*[W]*
		*DE [ml·min*^*-1*^*/WR*_*peak*_*]*
		
	**Muscular strength**	**Maximal force** (Hand-held dynamometer)
		*Grip strength [N]*
		*Elbow flexors & extensors [N]*
		*Shoulder abductors [N]*
		*Knee flexors & extensors [N]*
		
	**Body composition**	**Height [m]** (Stadiometer)
		**Weight [kg]** (Electronic scale)
		**Waist circumference [cm]** (Measuring tape)
		**Skinfold thickness** sum of four thicknesses **[mm]** (Caliper)
		
	**Flexibility**	**Range of motion** (Goniometer)
		*Wrist dorsal & palmar extension [degrees]*
		*Elbow flexion & extension [degrees]*
		*Shoulder anteflexion [degrees]*
		*Knee flexion & extension [degrees]*
		*Ankle plantar & dorsal extension [degrees]*
		
**Physical activity**		**Objectively assessed physical activity** (Accelerometer)
		**Self-reported physical activity** (Activity questionnaire, 16 items)
		*Subscale Dutch Standard Questionnaire for Activity (12 items)*
		*Subscale FIT Norm (2 items)*
		*Subscale Dutch Activity Norm (2 items)*
		
**Health-related quality of life**		**Health-related quality of life** (Burn Outcomes Questionnaire, 53 items)
		*Subscale upper extremity function (7 items)*
		*Subscale physical function and sports (6 items)*
		*Subscale transfers and mobility (5 items)*
		*Subscale pain (2 items)*
		*Subscale itch (2 items)*
		*Subscale appearance (4 items)*
		*Subscale compliance (5 items)*
		*Subscale satisfaction with current state (7 items)*
		*Subscale emotional health (4 items)*
		*Subscale family disruption (5 items)*
		*Subscale parental concern (3 items)*
		*Subscale school re-entry (3 items)*
		
**Fatigue**		**Fatigue** (PedsQL Multidimensional Fatigue Scale, 18 items)
		*Subscale General Fatigue score (6 items)*
		*Subscale Sleep/Rest Fatigue score (6 items)*
		*Subscale Cognitive Fatigue score (6 items)*

#### Physical fitness

Physical fitness will be objectively and quantitatively assessed, measuring four components of health-related physical fitness [19], i.e. muscular strength, cardiorespiratory endurance, body composition and flexibility, see Table 3. All measurements will be performed by the first author and using the same instruments to prevent bias.

• Cardiorespiratory endurance

Aerobic exercise capacity will be measured using an incremental maximal exercise test on an electronically braked cycle ergometer (Lode Corrival, Lode, ProCare BV, Groningen, The Netherlands). The subjects will start with a three minute warming-up of unloaded cycling, after which the work rate (WR [W]) will increase according to the Godfrey protocol [20]. While cycling the subject will breathe through a small face mask with low dead space, adapted to his/her face, which is connected to a calibrated mobile metabolic cart (Cortex Metamax, Cortex Medical, Leipzig, Germany). From the expired gas, minute ventilation (*V*E), oxygen uptake (*V*O_2_), carbon dioxide output (VCO_2_) and RER (respiratory exchange ratio= VCO_2/_*V*O_2_) will be calculated (corrected for dead space of the mask) by a computer connected to the gas analysis system. Additionally, saturation of the blood and heart rate (in beats per minute: bpm) will be monitored continuously during the exercise test, using pulse-oximetry and a heart rate monitor, respectively. A heart rate (HR_peak_) above 180 bpm and/or a RER_peak_ above 1.01 will be regarded as criteria for maximal effort [21,22].

The main outcome variables will be the VO_2peak_ [ml·min^-1^]: the average volume of the absolute oxygen uptake during the last 30 seconds of the test; and the VO_2peak_/kg: the absolute VO_2peak_ divided by body mass. The main outcome variables will be the VO_2peak_ [ml·min^-1^]: the average volume of the absolute oxygen uptake during the last 30 seconds of the test; and the VO_2peak_/kg: the absolute VO_2peak_ divided by body mass, see Table 3. Other outcomes are the highest work rate achieved (WR_peak_) and %HR01, %HR02, representing recovery of heart rate during the first and second minute in rest after the test. Delta efficiency, the oxygen uptake to work rate slope, will be calculated afterwards by dividing the increase in oxygen from rest to VO_2peak_ by WR_peak_.

The outcomes will be compared with age- and sex-matched Dutch reference values [22].

• Muscular strength

Maximal isometric muscle strength (in Newtons: N) will be quantified with a calibrated hand-held dynamometer (Citec, type CT 3001, CIT Techniques, Groningen, The Netherlands), see Table 3. The shoulder abductors, knee flexors and extensors, elbow flexors and extensors and grip strength will be tested three times with at least 30s interval, at the dominant side of the body. The test protocols are adopted from Beenakker et al. (2001) and for grip strength from Wind et al. (2009) to enable comparison with age- and sex-matched Dutch reference values from those studies [23,24]. The highest force generated will be taken as the final score. In case of burn scars over or close to a joint, the involved muscle groups will be tested at both sides of the body, so that scores of the burned and non-burned side can be compared.

• Body composition

The outcome variables for body composition are height, weight, body mass index (BMI), waist circumference and skinfold thicknesses (Table 3).

Body height will be measured to the nearest centimeter with a “wall”-mounted stadiometer, and body weight will be measured with an electronic scale, to the nearest 100 gram. Calculated BMI values $\left(\text{kg},/,{\text{m}}^{2}\right)\left(\frac{\text{weight}\left(\text{kg}\right)}{{\left(\text{height},\left(\text{m}\right)\right)}^{2}}\right)$ will be compared with age- and sex-matched reference values from TNO [25].

Waist circumference will be measured with a tape (measure tape 201, Seca, Hamburg, Germany), at the smallest area between the lowest rib and the hip-bone. Scores will be compared to Dutch values obtained from TNO [26].

Skinfold thicknesses will be assessed using a Harpenden skinfold caliper (Baty International, West Sussex, England) according to the protocol by Gerver & De Bruin (1996) which enables comparison with the age- and sex-matched reference values presented in that study [27]. At each site three measures will be taken, from which an average score will be calculated and used as the final score. When a site of measurement is scarred skinfold measurement will be not performed at that site.

• Flexibility

Passive joint range of motion (in degrees) will be measured around wrist, elbow, shoulder, knee and ankle on the dominant side of the body with a goniometer (Gollehon extendable goniometer 01135, Lafayette Instrument, Lafayette, U.S.A.), see Table 3. In case of burn scars over or surrounding a specific joint, this joint will be measured at both sides of the body to enable comparison. Standardized measurement protocols and, for comparison, Dutch reference values will be used [28]; personal communications].

#### Physical activity

Physical activity will be monitored using objective, quantitative assessment as well as self-report (Table 3). For the objective measure of daily physical activity accelerometry will be applied. The ActiGraph accelometer (GT3X+, ActiGraph, Pensacola, Florida, U.S.A.) will be worn on the right hip for seven consecutive days to monitor physical activity and gives activity counts as output [29]. Additionally, subjects will briefly report their activities as well as for which period during the day the accelerometer is worn.

To gain insight in the habitual physical activity and participation in sports of children after burns an activity questionnaire is used. The questions covering the Dutch physical activity guidelines (‘Dutch Norm for Healthy Activity’ and ‘Fit’-norm [30,31]) are put together with the ‘Standard Questionnaire for Activity’ [31,32] (Table 3). The first two enable comparisons with Dutch norms, while the latter includes questions about transfer activities, sport participation and sedentary behavior (TV, gaming). A parental version for parents of children aged 6 to 11 and a version for children up from the age of 12 will be used.

#### Health-related quality of life

As HRQoL is an experienced state, it will be subjectively measured using a child- and burn-specific questionnaire: the Dutch version of the American Burn Association/Shriners’ Hospital for Children Burn Outcomes Questionnaire (BOQ) is used, which had proven to be a feasible, reliable and valid instrument, also in the Dutch population of children with burns [33]. The BOQ assesses 12 functional and psychosocial domain scales and, for example, includes items on comorbidity, functioning at school and participation in leisure time activities, see Table 3. The parental proxy version will be applied for children up to and including 11 years of age and the adolescent version will be used for children aged 12 and older.

#### Fatigue

Perceived fatigue will be measured with the Dutch version of the 18-item PedsQL Multidimensional Fatigue Scale [34]. This scale, designed to measure fatigue in pediatric patients, comprises three subscales: the General Fatigue, Sleep/Rest Fatigue and Cognitive Fatigue (Table 3). The Dutch version has recently demonstrated adequate feasibility, reliability and validity [35]. Age-specific versions of both child and parent reports will be used for the age groups 6–7 years, 8–12 years and 13–18 years.

### Data analyses

Descriptive statistics will be used to present subjects’ demographic and burn characteristics and also for quantitative data attained from fitness tests and physical activity monitoring. To find out whether differences in physical fitness exist between children after burn injury and the general population of Dutch children, data from the fitness assessments will be compared to reference values from the literature. To this end, data will be transformed into Z-scores based on the reference values and independent t-tests will be used.

To determine associations between the physical fitness (primary outcome parameter: VO_2peak_) and the amount of physical activity (total activity counts), Pearson correlation coefficients will be calculated. Associations between the individual characteristics and the outcome parameters for fitness, activity, HRQoL and fatigue will be calculated by Pearson or Spearman’s correlation coefficients, depending on the scale of measurement and on the distribution of the data. Regression and/or modeling may additionally be used. IBM SPSS Statistics version 20 will be used for analyses.

## Discussion

The current paper describes the rationale, design and methods of a cross-sectional study concerning physical fitness and physical activity in children after burn injury. The study aims to determine the levels of physical fitness and physical activity in children after a burn injury and to place these findings in a multidimensional context, taking into account the experienced HRQoL and fatigue after burns, as well as several demographic and burn characteristics.

As objective measurements are chosen where possible and as the study procedures are standardized and adopted from the studies of which Dutch reference values will be used, we feel that the presented design and methods are strong. All physical fitness assessments will be done by the same rater, using the same instruments and will take place at the same location. Moreover, the use of the mobile exercise lab strongly minimizes the inconvenience of participation and with that is expected to maximize the participation rate. The study population is expected to form a good representation of the general population of children and adolescents with burns.

New insights gained from this study can stimulate and guide the improvement of rehabilitation protocols to facilitate a timely and optimal functional recovery of children after burns. As the study is done in close collaboration with the Association of Dutch Burn Centers knowledge transfer is evident and chances of implementation are promising. Overall, this unique study will contribute to a better understanding of the consequences of pediatric burn injury on physical functioning on the long term and to improvement of outcomes for people after burn injury in future.

## Abbreviations

BOQ: Burns Outcomes Questionnaire; Bpm: Heartbeats per minute; VCO_2_: Carbon dioxide output; DE: Delta efficiency; %HR01: %HR02; Recovery of heart rate during the second minute in rest after the test; HR_peak_: Peak heart rate; HRQoL: Health-related quality of life; PedsQL-MFS: PedsQL Multidimensional Fatigue Scale; TBSA: Total body surface area; VE: Minute ventilation; VO_2peak_: Peak volume oxygen uptake; WR_peak_: Peak work rate on cycle ergometer.

## Competing interests

The authors declare that they have no competing interests.

## Authors’ contribution

MKN and LMD developed the study design, for which GIJMB, TT and MVB used their expertise to advice on the measures chosen in the protocol. LHVVDW and LJM substantially contributed to the realization of this paper. All authors have critically revised the manuscript. They all read and approved the final version for publication.

## Pre-publication history

The pre-publication history for this paper can be accessed here:

http://www.biomedcentral.com/1471-2431/12/195/prepub
